# RelB-Dependent Stromal Cells Promote T-Cell Leukemogenesis

**DOI:** 10.1371/journal.pone.0002555

**Published:** 2008-07-02

**Authors:** Nuno R. dos Santos, Maryvonne Williame, Stéphanie Gachet, Françoise Cormier, Anne Janin, Debra Weih, Falk Weih, Jacques Ghysdael

**Affiliations:** 1 Institut Curie, Centre de Recherche, Orsay, France; 2 CNRS UMR146, Orsay, France; 3 INSERM Unité 728, Université Paris VII, Hôpital Saint-Louis, Paris, France; 4 Leibniz-Institute for Age Research – Fritz-Lipmann-Institute (FLI), Jena, Germany; Northwestern University, United States of America

## Abstract

**Background:**

The Rel/NF-κB transcription factors are often activated in solid or hematological malignancies. In most cases, NF-κB activation is found in malignant cells and results from activation of the canonical NF-κB pathway, leading to RelA and/or c-Rel activation. Recently, NF-κB activity in inflammatory cells infiltrating solid tumors has been shown to contribute to solid tumor initiation and progression. Noncanonical NF-κB activation, which leads to RelB activation, has also been reported in breast carcinoma, prostate cancer, and lymphoid leukemia.

**Methodology/Principal Findings:**

Here we report a novel role for RelB in stromal cells that promote T-cell leukemogenesis. RelB deficiency delayed leukemia onset in the TEL-JAK2 transgenic mouse model of human T acute lymphoblastic leukemia. Bone marrow chimeric mouse experiments showed that RelB is not required in the hematopoietic compartment. In contrast, RelB plays a role in radio-resistant stromal cells to accelerate leukemia onset and increase disease severity.

**Conclusions/Significance:**

The present results are the first to uncover a role for RelB in the crosstalk between non-hematopoietic stromal cells and leukemic cells. Thus, besides its previously reported role intrinsic to specific cancer cells, the noncanonical NF-κB pathway may also play a pro-oncogenic role in cancer microenvironmental cells.

## Introduction

The Rel/NF-κB transcription factors function in multiple biological processes, including development, immunity, inflammation, and response to cellular stress [1]. NF-κB subunits are often activated in solid or hematological malignancies as the result of rearrangements/mutations in their genes or in genes encoding components of the NF-κB signaling pathway, persistent autocrine or paracrine stimulation through specific cell surface receptors, or viral or cellular oncoprotein activity (for review see [2], [3]). NF-κB activation in cancer cells has been shown to activate genes involved in cell survival, proliferation, angiogenesis, invasion, and chemoresistance being therefore an important target for cancer therapy. Recently, an important function for the canonical NF-κB pathway in inflammatory cells infiltrating several types of solid tumors has been brought to light. NF-κB activation in those cells leads to the production of cytokines, growth factors, and angiogenic factors that promote malignant conversion and progression (for review see [3]).

The NF-κB proteins are transcriptional regulators that bind cognate DNA elements as homo- or heterodimers. NF-κB activity is controlled by interaction with IκB (inhibitor of NF-κB) proteins and only when these are degraded by the proteasome, following serine phosphorylation by IκB kinases (IKK) and ubiquitination, are NF-κB dimers released. The NF-κB/Rel family comprises five members (RelA, RelB, c-Rel, p50/p105, and p52/p100) sharing the conserved Rel homology domain, which is responsible for DNA binding, nuclear localization, dimerization, and IκB binding. In contrast to RelA (p65), RelB, and c-Rel, the p50 and p52 proteins, which derive from proteolytic processing of the p105 and p100 precursor proteins, respectively, lack transactivation domains. The p50 and p52 proteins act thus as transcriptional repressors, except when forming heterodimers with other NF-κB members or when interacting with other transcriptional activators, such as the Bcl3 protein (for review see [1]).

Two main NF-κB activation pathways have been identified [1]. The canonical NF-κB activation pathway, which is triggered by an array of stimuli such as proinflammatory cytokines, antigen receptors, Toll-like receptors, and cellular stress, relies on IKKβ (IKK2)/IKKγ (NEMO)-dependent IκB phosphorylation and degradation and results in RelA and/or c-Rel activation. Disruption of the canonical pathway in immune cells impairs innate and acquired immune responses in a cell-autonomous or non cell-autonomous manner (for review see [4]). The noncanonical NF-κB activation pathway, which can be activated by specific members of the TNF receptor family (e.g., lymphotoxin β receptor [LTβR] and BAFF receptor [BAFF-R]) depends on IKKα (IKK1) and NIK kinase activity but not on IKKβ or IKKγ [1]. Upon stimulation, IKKα phosphorylates p100 on C-terminal serine residues and induces its ubiquitin-dependent processing to generate p52. When released from p100 sequestration, p52:RelB, p50:RelB, and, as recently shown, p50:RelA dimers shuttle to the nucleus to activate transcription of specific target genes [5]–[8]. Disruption of the noncanonical pathway also affects immune cell function, impairing either lymphoid organogenesis due, at least in part, to defective LTβR signaling, or mature B cell function and maintenance due to defective BAFF-R signaling [9]. Furthermore, inactivation of the noncanonical pathway breaks down central tolerance as a result of impaired generation of medullary thymic epithelial cells (mTEC), which are essential for negative selection of autoreactive T cells [9]–[11].

Most studies in human lymphoid leukemia and lymphoma have identified canonical NF-κB activation in leukemic cells. For example, NF-κB activation is frequently observed in Hodgkin's lymphomas due to activation of the CD30, CD40, and RANK receptors or due to inactivating mutations in the IκBα-encoding gene [12]. Activation of p50 homodimers and p50:RelA heterodimers was detected in all major subtypes of human acute lymphoblastic leukemia (ALL) [13]. The v-*rel* oncogene, the retroviral counterpart of c-*rel*, induces aggressive leukemia/lymphoma in chicken and transgenic mice [14]. Canonical NF-κB activity was also found in T-ALL induced in mice following expression of a Tal1 transgene or of intracellular Notch1 (Notch1-IC) oncogenic protein [15], [16]. Finally, both the canonical and noncanonical NF-κB pathways were found to be activated by viral oncoproteins, in particular the HTLV1-encoded Tax protein in adult T-cell leukemia and the EBV-encoded LMP1 protein in B-cell lymphoma [12].

Several reports indicate that the noncanonical NF-κB pathway is also activated in specific subtypes of lymphoid leukemia and lymphoma (for review see [9], [12], [17]). Chromosomal translocations disrupting the *Nfkb2* gene that generate truncated p100 proteins and constitutive processing of p100 to p52 were identified in cutaneous T-cell lymphoma, and, more rarely, in B-cell non-Hodgkin lymphoma, chronic lymphocytic leukemia, and multiple myeloma [9], [12], [17]. Transgenic expression of a truncated p100 protein led to the development of B-cell lymphomas in mice, thus demonstrating the oncogenic potential of *Nfkb2* mutations [18]. Recently, genetic alterations in components of the noncanonical and canonical NF-κB pathways (e.g., *NIK*, *TRAF3*, *CYLD*, *BIRC2/BIRC3*, *CD40*, *NFKB1*, or *NFKB2*) resulting in their activation have been identified in multiple myeloma [19], [20].

In the present report we assessed the role of NF-κB proteins in a transgenic mouse model for human T-ALL induced by the TEL-JAK2 fusion protein [21], [22] and uncovered a specific role for RelB in T-cell leukemia development. Using RelB knockout mice we found that RelB assisted TEL-JAK2-induced T-cell leukemogenesis. Interestingly, bone marrow chimeric mouse experiments showed that RelB is not required in the hematopoietic compartment but plays a role in radio-resistant stromal cells to favor leukemia onset and increase disease severity.

## Results

### TEL-JAK2 leukemic cells display RelA and RelB activation

**Figure 1:**
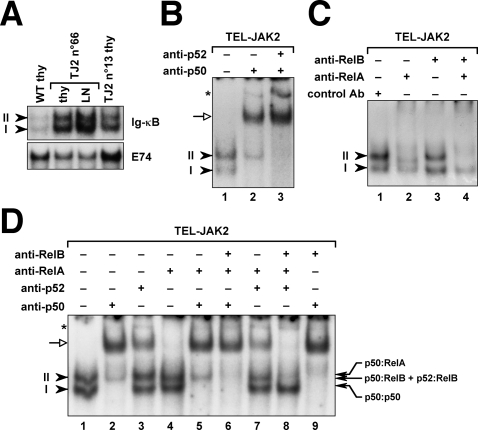
Activation of NF-κB transcription factors in TEL-JAK2 leukemic cells. (A) Nuclear extracts obtained either from freshly isolated wild-type control thymocytes (WT thy) or from thymus (thy) or lymph node (LN) leukemic cells from two representative TEL-JAK2 (TJ2) diseased mice (n°66 and n°13) were analyzed by electrophoretic mobility shift assays (EMSA) using the NF-κB-binding Ig-κB oligonucleotide probe. Similar results were obtained with more than 20 primary leukemic samples. Bands labeled *I* and *II* (arrowheads) indicate specific NF-κB complexes as assessed by competitive EMSA (data not shown). An Ets-specific probe (E74) was used for nuclear extract normalization. (B–D) Antibody supershift analysis of Ig-κB-bound complexes using the indicated antibodies was performed on leukemic nuclear extracts from individual TEL-JAK2 tumors (tumor n°1284 in panel B and D; tumor n°38 in panel C). Open arrows indicate supershifted complexes, filled arrows identify the different NF-κB complexes, and the asterisk indicates a nonspecific band. p50, p52, RelA, and RelB DNA-binding activity in TEL-JAK2 leukemic cells was also detected using a palindromic NF-κB probe derived from the IL2Rα promoter, indicating that the previous results were not biased by differential DNA recognition by NF-κB proteins.

To assess the NF-κB activity status in leukemic T cells from EμSRα-TEL-JAK2 transgenic mice, we performed electrophoretic mobility shift assays (EMSA) on nuclear extracts using an NF-κB-specific oligonucleotide probe. Nuclear extracts from TEL-JAK2 tumor cells showed significantly higher levels of NF-κB DNA-binding activity, visible as two bands with different mobility, as compared to control thymocyte nuclear extracts (Figure 1A). To determine which NF-κB members were activated in TEL-JAK2 leukemic cells, we pre-incubated nuclear extracts with specific NF-κB antibodies that either supershift or inhibit protein/DNA complexes. A p50/NF-κB1 antibody quantitatively supershifted band *I* and most of band *II* in TEL-JAK2 leukemic cells (Figure 1B, compare lanes 1 and 2). The remaining NF-κB activity in band *II* was inhibited by a p52/NF-κB2 antibody (Figure 1B, lane 3), indicating the presence of both proteins in different complexes. Band *II* complexes also included RelA and RelB since the RelA antibody inhibited a slowly migrating complex within band *II* (Figure 1C, lane 2 and Figure S1A, lane 3), leaving intact a faster migrating complex recognized by the RelB antibody (Figure 1C, lanes 3 and 4 and Figure S1A, lane 4). No c-Rel DNA-binding activity was found in TEL-JAK2 leukemic cells (Figure S1A, lane 5), although this was easily detected in thymocytes stimulated by phorbol 12-myristate 13-acetate (PMA) plus ionomycin (Figure S1B, lane 4).

To identify the NF-κB dimers activated in TEL-JAK2 leukemic cells, we performed supershift analyses combining different antibodies. Combination of RelA and p50 antibodies yielded the same migration pattern as that observed with p50 antibody alone (Figure 1D, compare lanes 2 and 5), indicating that RelA heterodimerized with p50. Combination of the RelB antibody with p50 and RelA antibodies inhibited the remaining complex (Figure 1D, compare lanes 5 and 6), which, given that RelB is unable to form homodimers [23], corresponds to p52:RelB heterodimers. Likewise, combination of the RelB antibody with p52 and RelA antibodies inhibited a complex that corresponded to p50:RelB heterodimers (Figure 1D, compare lanes 7 and 8). In sum, supershift analyses discerned the presence of p50:p50 homodimers as well as p50:RelA, p50:RelB, and p52:RelB heterodimers in TEL-JAK2 leukemic cells.

### *Nfkb1* deficiency does not affect TEL-JAK2-induced T-cell leukemia development

**Figure 2:**
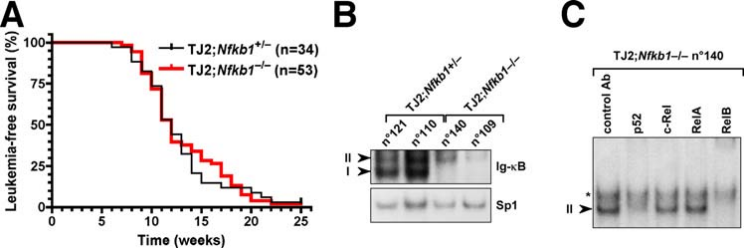
*Nfkb1* deficiency did not impair TEL-JAK2-induced T-cell leukemogenesis. (A) Kaplan-Meier leukemia-free survival curves for EμSRα-TEL-JAK2 transgenic mice deficient or not for the *Nfkb1* gene. No statistically significant differences were observed. The number of mice in each group is given between parentheses. (B) Leukemic cell nuclear extracts from representative TEL-JAK2;*Nfkb1* ^+/−^ (mice n°110 and n°121) and TEL-JAK2;*Nfkb1* ^−/−^ (mice n°109 and n°140) mice were analyzed by EMSA using the Ig-κB and Sp1 probes. *I* and *II* indicate specific DNA-bound NF-κB complexes. Note the absence of band I (p50 homodimers) in *Nfkb1* ^−/−^ samples. (C) Antibody supershift analysis using the indicated antibodies on Ig-κB-bound complexes derived from representative TEL-JAK2;*Nfkb1* ^−/−^ (mouse n°140) leukemic cells. Band *II* corresponds to p52/RelB DNA-bound heterodimers. The asterisk indicates a non NF-κB-specific band.

To assess the role of NF-κB proteins in TEL-JAK2-induced leukemogenesis, we bred EμSRα-TEL-JAK2 mice with mice deficient for specific NF-κB genes. To prevent p50-containing complex formation, we first bred EμSRα-TEL-JAK2 mice with *Nfkb1* knock-out mice, which do not express the NF-κB1 proteins p50 and its precursor p105 and do not show any thymocyte maturation defects [24]. EμSRα-TEL-JAK2;*Nfkb1* ^−/−^ and EμSRα-TEL-JAK2;*Nfkb1* ^+/−^ littermate mice developed T-cell leukemia with full penetrance and similar latency (Figure 2A; median survival of 12 weeks). In addition, *Nfkb1*-deficient leukemic cells presented a cell surface marker phenotype (expression of variable levels of CD4, CD8, CD24, CD25, and TCRβ/CD3ε) similar to that of *Nfkb1*-proficient cells (data not shown) and characteristic of transgenic TEL-JAK2 leukemia [21], [25].

EMSA analyses showed that the TEL-JAK2;*Nfkb1* ^−/−^ leukemic cells displayed severely reduced NF-κB activity and, as expected, did not display any p50 DNA-binding activity, as evidenced by the lack of p50 homodimers (band *I*) (Figure 2B). Interestingly, no RelA DNA-binding activity was detectable in nuclear extracts from TEL-JAK2;*Nfkb1* ^−/−^ leukemic cells (Figure 2C). This was not due to an intrinsic inability to activate RelA in these cells, since TEL-JAK2;*Nfkb1* ^−/−^ leukemic cells induced p52:RelA and RelA:RelA DNA-bound dimers upon in vitro treatment with PMA plus ionomycin (Figure S2). This result suggests that activation of p50 and/or RelA does not play a nonredundant role in TEL-JAK2 leukemogenesis. The only DNA/protein complexes identified in TEL-JAK2;*Nfkb1* ^−/−^ leukemic cells were p52:RelB heterodimers, since formation of this complex was inhibited by antibodies against either p52 or RelB, but not by p50-, c-Rel-, or RelA-specific antibodies (Figure 2C and data not shown).

### Generation of viable *Relb*-deficient mice

Since TEL-JAK2;*Nfkb1* ^−/−^ leukemic cells only presented constitutive p52:RelB activity, we set out to evaluate the role of RelB in TEL-JAK2-induced leukemogenesis by generating *Relb*-deficient TEL-JAK2 transgenic mice. *Relb*-deficient mice present fatal T-cell-dependent multiorgan inflammation, a phenotype resulting from the absence of mTEC, which are essential for negative selection of autoreactive T cells [26]–[28]. To generate viable *Relb*-deficient mice, these were bred with *Tcra*-deficient mice, which do not express the αβ T-cell receptor (TCR) and therefore lack mature T cells. The resultant *Tcra* ^−/−^;*Relb* ^−/−^ mice were born at expected Mendelian ratios and lived without external signs of inflammation or other abnormal phenotype, with the exception that female mice failed to nurse their pups. Histological analysis showed no inflammatory infiltrates in liver, lungs, and skin of *Tcra* ^−/−^;*Relb* ^−/−^ mice (Figure S3A and data not shown), demonstrating that *Tcra* deficiency rescued the inflammatory phenotype observed in *Relb*-deficient mice [26]–[28]. In line with previous findings [28], we observed that mature T-cell deficiency in *Relb*-null mice prevented splenomegaly and splenic myeloid hyperplasia (data not shown).

**Figure 3:**
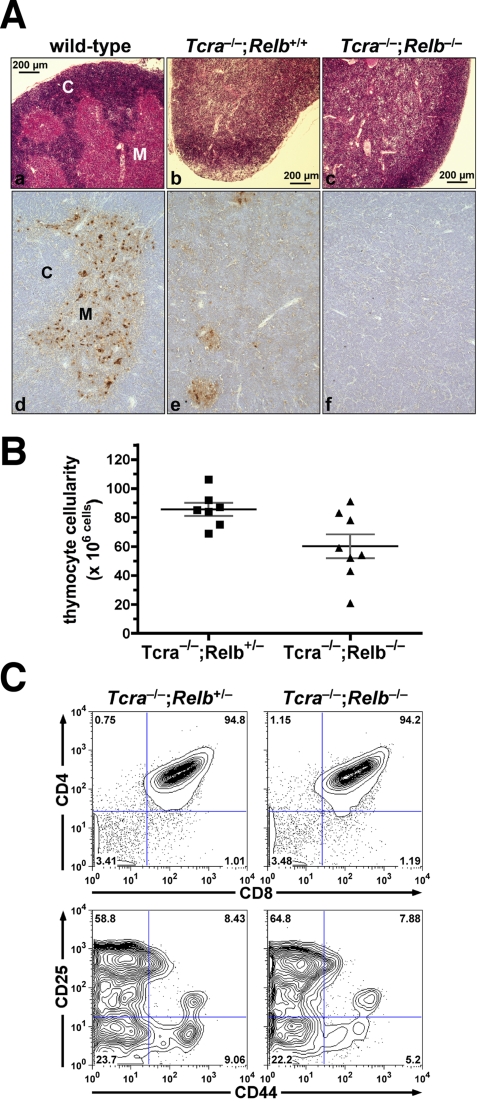
*Tcra*;*Relb* double deficient mice present defective thymic microarchitecture without impairment of double positive thymocyte development. (A) Thymi from mice with the indicated genotypes were stained by H&E (panels a–c) or with UEA-1 lectin, a cell surface marker of medullary thymic epithelial cells (d–f). Note the clear distinction between medulla (M) and cortex (C) in the wild-type thymus. (B) Thymocyte total cell counts were plotted for *Tcra* ^−/−^;*Relb* ^+/−^ (n = 7) and *Tcra* ^−/−^;*Relb* ^−/−^ (n = 8) mice aged from 12–16 weeks. (C) CD4, CD8 cell surface immunostaining of total thymocytes (top panels) and CD25, CD44 staining of Thy1.2^+^, CD4/CD8 double negative cells (bottom panels) of representative mice of the indicated genotypes.

*Relb*-deficient mice present defective development of thymus and secondary lymphoid organs [26], [27], [29]. The defective lymph node development linked with RelB deficiency was not rescued by mature T-cell deficiency (Figure S3B), showing that it is a non-T-cell-dependent phenotype. In contrast, *Tcra* ^−/−^;*Relb* ^−/−^ mice presented thymi of size similar to that of *Tcra* ^−/−^;*Relb* ^+/+^ littermates (data not shown), confirming the notion that the severe thymic atrophy seen in *Relb*-null mice is mainly due to T-cell dependent multiorgan inflammation [30]. In contrast to wild-type thymi, *Tcra* ^−/−^;*Relb* ^+/+^ thymi presented reduced and disorganized medulla with few scattered *Ulex europaeus* agglutinin 1 (UEA-1)-positive mTECs (Figure 3A), a phenotype caused by the absence of mature T-cell crosstalk with the thymic stroma [11], [31], [32]. *Tcra* ^−/−^;*Relb* ^−/−^ thymi, like *Relb* ^−/−^ thymi [26], [27], [31], lacked distinctive medulla and did not present any UEA-1^+^ mTECs (Figure 3A). Despite a 1.4-fold reduction in thymocyte cellularity in *Tcra* ^−/−^;*Relb* ^−/−^ mice as compared to *Tcra* ^−/−^;*Relb* ^+/−^ mice (Figure 3B), early thymocyte development proceeded similarly in both *Relb*-deficient and *Relb*-proficient mice. Indeed, both groups of mice presented similar proportions of CD4^−^CD8^−^ double negative (DN), CD8 immature single positive, and CD4^+^CD8^+^ double positive (DP) thymocytes (Figure 3C and data not shown). As expected, due to the *Tcra* mutation no CD4 and CD8 single-positive (SP) cells were observed in the two groups of mice. The proportion of DN subsets, as defined by CD25 and CD44 expression, and TCRγδ T-cell development was also unaffected by RelB deficiency in *Tcra* ^−/−^;*Relb* ^−/−^ mice (Figure 3C and Figure S4).

### *Relb* deficiency delays the onset of TEL-JAK2-induced T-cell leukemia

**Figure 4:**
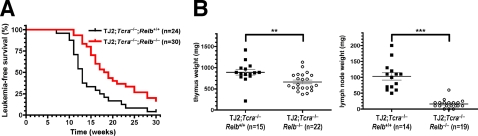
*Relb* deficiency delays the onset of TEL-JAK2-induced T-cell leukemia. (A) Kaplan-Meier leukemia-free survival curves for EμSRα-TEL-JAK2;*Tcra* ^−/−^ transgenic mice deficient or not for the *Relb* gene are significantly different (log-rank test, *P* value<0.01). The number of mice in each group is given between parentheses. (B) Thymus and lymph node weights were plotted for leukemic *Relb* ^+/+^ and *Relb* ^−/−^ TEL-JAK2;*Tcra* ^−/−^ mice. **, *P* value<0.01; ***, *P* value<0.001 (unpaired *t*-test). The number of mice analyzed is given between parentheses.

Our previous studies have shown that breeding of TEL-JAK2 transgenic mice on a *Tcra*-deficient background does not delay leukemia onset and incidence [25], although TEL-JAK2;*Tcra* ^−/−^ leukemic cells showed reduced RelA DNA-binding activity as compared to *Tcra*-proficient leukemic cells while maintaining a similar level of RelB DNA binding activity (Figure S5). In contrast, when EμSRα-TEL-JAK2 mice were bred on a *Tcra* ^−/−^;*Relb* ^−/−^ background, we found that these mice developed T-cell leukemia with statistically significant delayed onset as compared to TEL-JAK2;*Tcra* ^−/−^;*Relb* ^+/+^ littermates (median survival of 18.5 and 13 weeks, respectively; *P*<0.01) (Figure 4A). Diseased mice from both groups presented leukemic cells in thymus, spleen, lymph nodes, bone marrow, liver, and lungs (Figure S6 and data not shown). However, TEL-JAK2;*Tcra* ^−/−^;*Relb* ^−/−^ mice presented significantly reduced tumor load in thymus and lymph nodes, as compared to *Relb*-proficient littermates (Figure 4B). Similar to *Relb*-proficient cells, *Relb*-deficient leukemic cells presented the variable levels of CD4, CD8, CD24, and CD25 cell surface markers that characterize TEL-JAK2 leukemic cells (Figure S7). TEL-JAK2;*Tcra* ^−/−^;*Relb* ^−/−^ leukemic cells showed similar p50:p50 and p50:RelA NF-κB DNA-binding activity as TEL-JAK2;*Tcra* ^−/−^;*Relb* ^+/−^ leukemic cells (data not shown), indicating that RelB inactivation did not lead to selection of leukemic cells displaying enhanced DNA-binding activity of other NF-κB family members. Together, these results reveal a non-redundant function for RelB in TEL-JAK2-induced leukemogenesis. However, because RelB deficiency affects all mouse tissues, these results do not distinguish whether RelB function is required intrinsically in the hematopoietic cells targeted by TEL-JAK2, and/or whether it is required in non-leukemic cells from the tumor microenvironment to support TEL-JAK2-induced leukemogenesis.

### *Relb* deficiency in the hematopoietic compartment does not affect TEL-JAK2-induced T-cell leukemogenesis

**Figure 5:**
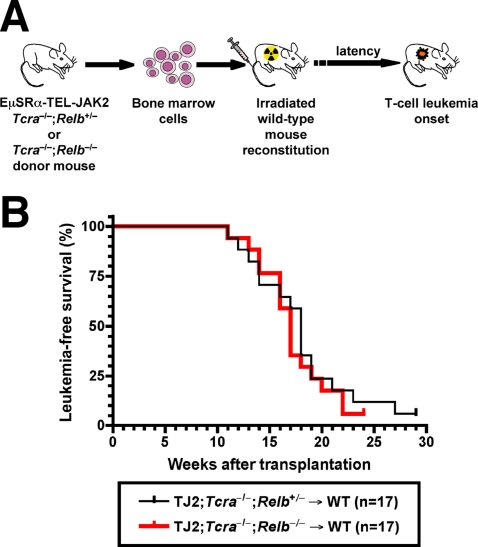
*Relb* deficiency in bone-marrow-derived hematopoietic cells does not affect the onset of TEL-JAK2-induced leukemia. (A) Schematic representation of the bone marrow adoptive transfer approach used to generate TEL-JAK2 transgenic mice lacking *Relb* in the hematopoietic compartment only. (B) Kaplan-Meier leukemia-free survival curves for wild-type (WT) recipient mice that received bone marrow from EμSRα-TEL-JAK2;*Tcra* ^−/−^ transgenic mice deficient or not for the *Relb* gene (3 donors of each genotype) show no statistically significant difference in survival. The number of mice analyzed in each group is given between parentheses.

To analyze whether the function of RelB in TEL-JAK2-induced leukemogenesis reflected an intrinsic role in the hematopoietic compartment, we generated bone marrow radiation chimeric mice. Bone marrow cells obtained from either TEL-JAK2;*Tcra* ^−/−^;*Relb* ^−/−^ or TEL-JAK2;*Tcra* ^−/−^;*Relb* ^+/−^ non-diseased mice were intravenously injected into wild-type (WT), lethally irradiated recipient mice (Figure 5A). Both groups of bone marrow chimeric mice developed T-cell leukemia with similar onset (median survival of 18 and 17 weeks for TEL-JAK2;*Tcra* ^−/−^;*Relb* ^+/−^→WT and TEL-JAK2;*Tcra* ^−/−^;*Relb* ^−/−^→WT mice, respectively) (Figure 5B). Diseased chimeric mice of both groups developed leukemia affecting the thymus, frequently in association with pleural effusion, lymph nodes, bone marrow, and occasionally spleen and liver (data not shown). No significant difference in lymphoid organ invasion and leukemic cell surface marker expression was observed between the two experimental groups (Figure S8 and data not shown). Of note, under these experimental conditions non-TEL-JAK2 transgenic *Tcra* ^−/−^;*Relb* ^−/−^ and *Tcra* ^−/−^;*Relb* ^+/−^ bone marrow donor cells were both capable to normally reconstitute the T-cell compartment when adoptively transferred into lethally irradiated syngeneic WT mice, as demonstrated by the similar proportions of DN and DP thymocytes and by the absence of mature CD4 or CD8 SP cells (Figure S9). Together, these results indicate that *Relb* deficiency in the hematopoietic compartment hampered neither normal T-cell development nor TEL-JAK2-induced T-cell leukemogenesis.

### *Relb* deficiency in radiation-resistant non-hematopoietic cells delays the onset of TEL-JAK2-induced T-cell leukemia

**Figure 6:**
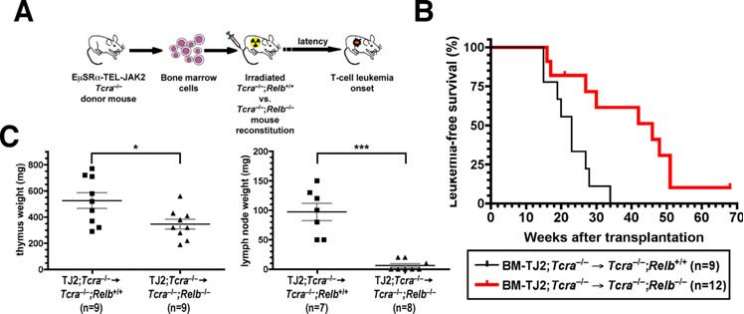
*Relb* deficiency in radiation-resistant non-hematopoietic cells delays the onset of TEL-JAK2-induced T-cell leukemia. (A) Schematic representation of the bone marrow adoptive transfer approach used to generate TEL-JAK2 transgenic mice lacking *Relb* in non-hematopoietic cells only. (B) Kaplan-Meier leukemia-free survival curves for *Tcra* ^−/−^ recipient mice deficient or not for the *Relb* gene that received bone marrow from EμSRα-TEL-JAK2;*Tcra* ^−/−^ transgenic mice were significantly different (log-rank test, *P* value<0.01). A mouse deceased from an unrelated cause was censored in the analysis (tick mark). (C) Thymus and lymph node weights were plotted for TEL-JAK2;*Tcra* ^−/−^→*Tcra* ^−/−^;*Relb* ^−/−^ and TEL-JAK2;*Tcra* ^−/−^→*Tcra* ^−/−^;*Relb* ^+/+^ diseased chimeric mice. *, *P* value<0.05; ***, *P* value<0.001 (unpaired *t*-test). The number of mice analyzed in each group is given between parentheses.

Since RelB is expressed in thymic and lymph node non-hematopoietic cells and since genetic experiments have shown that RelB plays an important role in shaping thymus and lymph node architecture during mouse development [26], [27], [33], we analyzed whether RelB could play a role in leukemogenesis through a hematopoietic cell-extrinsic mechanism. First, we assessed the ability of *Tcra* ^−/−^;*Relb* ^−/−^ mice cells to support normal development of *Relb* ^+/+^ T cells. Therefore, *Tcra* ^−/−^;*Relb* ^+/+^ bone marrow cells were adoptively transferred into lethally irradiated *Tcra* ^−/−^;*Relb* ^−/−^ (*Tcra* ^−/−^→*Tcra* ^−/−^;*Relb* ^−/−^) or *Tcra* ^−/−^;*Relb* ^+/−^ (*Tcra* ^−/−^→*Tcra* ^−/−^;*Relb* ^+/−^) recipient mice. Expression of CD4, CD8, CD25, and CD44 showed that both groups of chimeric mice presented normal DN and DP thymocyte development (Figure S10A). No significant difference in thymocyte cellularity was observed between *Tcra* ^−/−^→*Tcra* ^−/−^;*Relb* ^−/−^ and *Tcra* ^−/−^→*Tcra* ^−/−^;*Relb* ^+/−^ mice (data not shown). In these experiments, donor female *Tcra* ^−/−^ bone marrow cells were adoptively transferred to male recipients, so efficient thymus reconstitution by donor cells could be ascertained by the presence of X-chromosome-specific genomic sequences, as detected by PCR, and the absence of Y-chromosome-specific sequences in thymocytes from chimeric recipient mice (Figure S10B). These results indicate that *Tcra* and *Relb* double deficiency in non-hematopoietic cells does not impair the development of DN and DP thymocytes, the T-cell compartment targeted by TEL-JAK2 in leukemogenesis [25]. Thus, bone marrow cells from non-diseased EμSRα-TEL-JAK2;*Tcra* ^−/−^ mice were intravenously injected into lethally irradiated *Tcra* ^−/−^;*Relb* ^−/−^ and *Tcra* ^−/−^;*Relb* ^+/+^ littermate recipient mice (Figure 6A). We found that *Tcra* ^−/−^;*Relb* ^−/−^ recipient mice reconstituted with TEL-JAK2;*Tcra* ^−/−^ bone marrow cells (TEL-JAK2;*Tcra* ^−/−^→*Tcra* ^−/−^;*Relb* ^−/−^) developed T-cell leukemia with delayed onset, as compared to the reconstituted *Tcra* ^−/−^;*Relb* ^+/+^ littermates (TEL-JAK2;*Tcra* ^−/−^→*Tcra* ^−/−^;*Relb* ^+/+^) (median survival of 46 and 23 weeks, respectively; *P*<0.01; Figure 6B). Two TEL-JAK2;*Tcra* ^−/−^→*Tcra* ^−/−^;*Relb* ^−/−^ mice even survived without leukemia for more than 60 weeks after transplantation (Figure 6B). PCR detection of the TEL-JAK2 transgene and the wild-type *Relb* allele in thymocytes from these mice showed effective donor bone marrow engraftment (data not shown). The two groups of mice developed severe dyspnea due to leukemic cell accumulation in the thymus and to pleural effusion containing leukemic cells (data not shown). Like the original TEL-JAK2 transgenic mice [21], diseased chimeric mice showed leukemic cell accumulation in the bone marrow, spleen, lungs, and liver (data not shown). Leukemic cells from both groups of mice expressed variable levels of CD4, CD8, CD24, and CD25 cell surface markers, characteristic of bona fide TEL-JAK2 T-cell leukemia (data not shown). However, in addition to a delayed onset, TEL-JAK2;*Tcra* ^−/−^→*Tcra* ^−/−^;*Relb* ^−/−^ diseased mice presented significantly reduced accumulation of leukemic cells in the thymus and lymph nodes, as compared to TEL-JAK2;*Tcra* ^−/−^→*Tcra* ^−/−^;*Relb* ^+/+^ mice (Figure 6C), indicating that the disease evolved more slowly in lymphoid organs from *Relb*-deficient chimeras. A similar difference in tumor burden was observed in an independent experiment comparing leukemia development between *Tcra* ^−/−^;*Relb* ^−/−^ and *Tcra* ^−/−^;*Relb* ^+/−^ recipients (Figure S11). Taken together, these results show that RelB has a specific, non-redundant function in radiation-resistant thymic and lymph node stromal cells that facilitates TEL-JAK2-induced T-cell leukemogenesis.

## Discussion

In the present study we have found that expression of the RelB transcription factor in non-hematopoietic, stromal cells promotes T-cell leukemogenesis induced by the TEL-JAK2 oncoprotein. These conclusions stem from two sets of data showing delayed leukemia onset in (*i*) TEL-JAK2 transgenic mice deficient in RelB, as compared to RelB-proficient littermates, and (*ii*) lethally irradiated RelB-deficient mice reconstituted with TEL-JAK2 transgenic bone marrow, as compared to similarly reconstituted RelB-proficient littermates. These results thus demonstrate that, in addition to their reported pro-oncogenic role intrinsic to leukemic cells in several mouse models [16], [34] and in human leukemia cell lines [12], NF-κB transcription factors contribute to T-cell leukemogenesis through the generation and/or maintenance of a proper stromal microenvironment.

*Relb* ^−/−^ mice develop lethal multiorgan inflammation shortly after birth [26], [27]. This disease is caused by the appearance of autoreactive mature T cells resulting from the absence of mTECs expressing Aire transcriptional regulator-dependent peripheral tissue antigens, which are important to establish central self-tolerance [26]–[28], [35]. The possibility that the delay in TEL-JAK2-induced leukemogenesis observed in RelB-deficient mice could be linked to the inflammatory phenotype of these mice can be excluded since our experiments were performed in the *Tcra* ^−/−^ genetic background, which prevents the development of autoreactive T cells. Indeed, in contrast to the reported RelB-deficient phenotype [26]–[28], no significant inflammatory infiltrates were observed in the organs of *Tcra* ^−/−^;*Relb* ^−/−^ mice.

Our previous results showed that TEL-JAK2 transforms immature DN and DP thymocytes [25]. Since DN and DP thymocyte development remained unaffected in the *Tcra* ^−/−^;*Relb* ^−/−^ thymic microenvironment, we exclude the possibility that the delay in TEL-JAK2-induced leukemogenesis in RelB-deficient mice was simply due to a reduction in cellular targets available for oncogenic transformation. Our results thus suggest that the RelB-dependent microenvironment contributes specifically to DN/DP thymocyte transformation by TEL-JAK2.

Mouse RelB deficiency results in impaired lymphoid organ microarchitecture, affecting to varying degrees the development of thymus, spleen, lymph nodes, and Peyer's patches [26], [27], [33]. Stromal defects in these lymphoid organs likely account for the observed delay in TEL-JAK2-induced leukemogenesis in *Tcra* ^−/−^;*Relb* ^−/−^ mice. Since RelB-deficient diseased mice (both TEL-JAK2;*Tcra* ^−/−^;*Relb* ^−/−^mice and TEL-JAK2;*Tcra* ^−/−^→*Tcra* ^−/−^;*Relb* ^−/−^ chimeric mice) presented reduced thymic and lymph node tumor load compared to RelB-proficient controls, we conclude that defects in these organs were responsible for the delay in leukemogenesis.

Although lymph node development is strongly affected by the lack of RelB, the direct cellular defects associated with RelB deficiency in this organ are as yet unknown. In contrast, it has been shown that RelB-deficient thymi lack a defined medulla and mTECs and show a strong reduction in CD80^+^DEC205^+^ dendritic cell (DC) numbers secondary to the defect in thymic architecture and mTECs [26], [27], [36]. Accordingly, *Tcra* ^−/−^;*Relb* ^−/−^ mice showed no discernable thymic medulla and no UEA-1^+^ mTECs. TCRα-deficient mice also show thymic architectural defects, with reduced and disorganized medulla and fewer UEA-1^+^ mTECs than wild-type mice [11], [31], [32]. These defects are restored by adoptive transfer of mature T cells [11]. The combined deficiency of *Tcra* and *Relb*, but not *Tcra* deficiency alone [25], delayed TEL-JAK2-induced leukemia onset, thus indicating that, contrary to the RelB-deficient thymic defects, those found in TCRα-deficient mice have no detectable impact on leukemia development. Gray et al [31] have recently shown that TCRα-deficient thymi lack MHC II^lo^/Ly51^−^ (mTEC^lo^) cells, while RelB-deficient thymi additionally lack MHC II^hi^/Ly51^−^ (mTEC^hi^) cells. It is thus tempting to speculate that specifically mTEC^hi^ cells assist TEL-JAK2-induced T-cell leukemogenesis, although we cannot exclude an additional requirement for a RelB-dependent function in other stromal cells including DCs or cortical thymic epithelial cells. Moreover, RelB-dependent thymic stromal cells may assist TEL-JAK2 leukemogenesis either directly, through cell-cell contact or paracrine growth factor stimulation, or indirectly by stimulating other stromal cells (e.g., DCs) to interact with leukemic cells.

The nature of the molecular signals emanating from the thymic or lymph node stroma that favor T-cell leukemia initiation or progression remains to be identified. It is likely that RelB activity in mTEC or lymphoid organ stromal cells induces the expression of genes that favor T-cell leukemogenesis. Proteins known to play a role in thymic function include cytokine/growth factors (e.g., IL-7, Kit ligand, Notch ligands, and Sonic Hedgehog), chemokines (e.g., Ccl19/Elc, Ccl21/Slc, Ccl25/Teck, and Cxcl12/Sdf-1), cell surface receptors (e.g., LTβR, RANK, and Notch-1 to -3) and adhesion molecules (e.g., ICAM-1, MAdCAM-1, and VCAM-1) [37], [38]. RelB DNA-binding activity can be stimulated by RANK and LTβR, two receptors coupled to NF-κB activation and shown to be important for thymic medulla and lymphoid organ formation [39]. Both receptors activate NF-κB through the canonical and noncanonical pathways, with RANK specifically requiring TRAF6. LTβR signaling in thymic mTECs and in lymph node DCs induces expression of *Ccl19* and *Ccl21* [40], [41], which are known RelB target genes [42], [43], and of these chemokines as well as MAdCAM1, ICAM-1, and VCAM-1 in lymph node stromal cell organizers [44]. Since TEL-JAK2 leukemic cells express the *Ccr7* transcript, encoding the receptor for the Ccl19 and Ccl21 chemokines (data not shown), and display cell surface expression of the ICAM-1 receptor LFA-1 (data not shown), it is tempting to speculate that these NF-κB signaling-dependent targets may play a role in TEL-JAK2-induced leukemogenesis.

Recent studies have shown that the composition of the thymic stroma is dynamic and modulated by particular stimuli (e.g., LTαβ, FGF family members, Wnt, and steroids) [45]. It is thus possible that leukemic T cells analogously induce qualitative and/or quantitative changes in thymic stromal populations. Our transcriptomic analysis showed higher expression levels of the LTα- and LTβ-encoding genes in TEL-JAK2 leukemic cells as compared to normal thymocytes ([25] and data not shown). It is therefore possible that LTα_1_β_2_ production by leukemic cells may modulate the thymic microenvironment in its favor through interaction with LTβR-expressing stromal cells and in this way contribute to leukemogenesis.

Our data cannot discriminate whether RelB-dependent stromal cells facilitate the initiation or the progression of T-cell leukemia, or both. Nevertheless, the limited tumor burden in thymus and lymph nodes of terminally ill TEL-JAK2;*Tcra* ^−/−^;*Relb* ^−/−^ and TEL-JAK2;*Tcra* ^−/−^→*Tcra* ^−/−^;*Relb* ^−/−^ mice suggests that the RelB-dependent thymic microenvironment favors the expansion of transformed leukemic cells. During normal T-cell development, *Tcra* ^−/−^;*Relb* ^−/−^ thymi presented a slight reduction in thymocyte cellularity as compared to *Tcra* ^−/−^;*Relb* ^+/−^ thymi, suggesting that both normal and leukemic T cell expansion is affected by RelB-dependent thymic stroma. The thymic medulla is a compartment where IL-7-dependent proliferation of mature T cells occurs before their export to the periphery [46], [47]. This compartment in RelB-proficient TEL-JAK2 mice could favor the expansion of leukemic cells in the thymus.

Stromal cells from lymphoid organs or from the bone marrow are important to sustain survival and proliferation of human leukemic cells. The survival of T-ALL primary cells in vitro is promoted by exogenous growth factors (e.g., IL-7) or by co-culture with bone marrow or thymic stromal cells [48]–[51]. Also, thymectomy was shown to prevent T-cell leukemia development induced by Ikaros deficiency in mice [52], further supporting an important role of the thymic microenvironment in T-cell leukemia development. That tumor microenvironmental-derived signals are required for the in vivo expansion of TEL-JAK2-induced leukemic cells is supported by the fact that these cells survived for over a week ex vivo but failed to proliferate under these conditions (data not shown).

TEL-JAK2 leukemic cells displayed NF-κB activity (p50, p52, RelA, and RelB), suggesting that, in addition to a noncell-autonomous role, NF-κB activation may also contribute cell-autonomously to TEL-JAK2 leukemia development or maintenance. Intrinsic canonical NF-κB activity has recently been shown to be important for Notch-induced murine T-cell leukemia, since disease development was inhibited by expression of the IκBα super-repressor mutant [16], [34]. This effect could be specific to these particular models of T-cell leukemia, since the IκBα super-repressor failed to affect Tal-1-induced T-ALL [15]. Transgenic co-expression of the IκBα super-repressor with TEL-JAK2 neither did inhibit NF-κB activity nor affected leukemia incidence or severity (N.d.S., Marie Körner, and J.G., unpublished data). Likewise, *Nfkb1* deficiency failed to affect TEL-JAK2-induced leukemogenesis. Interestingly, TEL-JAK2;*Nfkb1* ^−/−^ leukemic cells did not present any RelA DNA-binding activity. In addition, RelA DNA-binding activity was reduced in TEL-JAK2;*Tcra* ^−/−^ compared to TEL-JAK2;*Tcra* ^+/−^ leukemic cells. These observations together with the fact that TCRα-deficiency did not affect TEL-JAK2 leukemia onset [25], indicates that RelA DNA-binding activity correlates neither with leukemia time of onset nor with disease progression. In addition, bone marrow adoptive transfer experiments showed that RelB deficiency in hematopoietic cells did not affect TEL-JAK2-induced leukemogenesis. These results indicate that cell-autonomous RelB expression is not essential for TEL-JAK2 leukemia initiation or maintenance. However, since NF-κB functional redundancy may exist among the different members of the NF-κB family and since complete NF-κB activation could not be experimentally achieved in TEL-JAK2 leukemic cells, we cannot rule out that NF-κB plays a cell-autonomous role, in addition to a non-cell-autonomous role, in TEL-JAK2-induced leukemogenesis.

The p52 and RelB proteins are activated by the noncanonical NF-κB activation pathway, which depends on NIK/IKKα-induced p100 processing. Accumulating evidence shows that the noncanonical NF-κB pathway is activated in the tumor cells of several human cancers, including B- and T-cell leukemia/lymphoma, mammary carcinoma, and prostate cancer [12], [17], [19], [20], [43], [53], [54]. The present results are however the first to uncover a role for RelB in the crosstalk between stromal and leukemic cells. Thus, the noncanonical NF-κB pathway may play a pro-oncogenic role both in tumor cells and in cells that compose the tumor microenvironment. In other settings, this property is shared by the canonical NF-κB activity which also plays an important role in activating both intrinsic genetic programs in cancer cells and the production of pro-oncogenic growth factors in cancer-associated stromal or inflammatory cells [3]. It will thus be important to understand how the thymic or lymphoid organ microenvironment assists T-cell leukemia development and to identify the leukemia-promoting factors that are dependent on noncanonical NF-κB activity in stromal cells. A better understanding of the crosstalk between leukemic T cells and stromal cells may pave the way for the development of new therapeutic strategies targeting this disease.

## Materials and Methods

### Mice

The EμSRα-*TEL-JAK2* transgenic mice (line 71) [21] were bred with *Tcra* (obtained from CNRS-CDTA; Orléans, France), *Relb* [27], and *Nfkb1* [24] knock-out mice on the C57BL/6 background. All mice were maintained under specific-pathogen-free conditions in the animal facility of the Institut Curie (Orsay, France). All experimental procedures were performed in strict accordance with the recommendations of the European Community (86/609/EEC) and the French National Committee (87/848) for the care and use of laboratory animals. All animal experiments were carried out under the supervision of J.G., who was authorized by the director of the Veterinary Services of the Préfecture de l'Essonne (agreement number 91-7). TEL-JAK2 transgenic mice were euthanized when terminally ill, due to either severe dyspnea caused by massive expansion of leukemic cells in the thymus or extreme weakness caused by leukemic dissemination to vital organs such as bone marrow, lung, and liver. Statistical analyses and survival curves were calculated using Prism 4 (GraphPad, San Diego, CA). Genomic PCR of the sex chromosome-localized *Jarid1c/Kdm5c* and *Jarid1d/Kdm5d* genes was performed as described by Clapcote and Roder [55].

### Bone marrow adoptive transfer experiments

Mouse bone marrow cells (BMC) were flushed out from hind legs. TEL-JAK transgenic BMC were obtained from nondiseased 4-week-old mice, as verified by the absence of leukemic cells (CD4^+^CD8^+^CD25^+^) in the thymus and bone marrow by flow cytometry. In adoptive transfer experiments, 2–3×10^6^ BMC were intravenously injected into lethally irradiated (8.125 Gy) mice using an IBL-637 (^137^Cs) γ-irradiator (CIS-BioInternational, Saclay, France).

### Cell isolation and culture

Single cell suspensions were prepared from lymphoid organs gently dissociated and filtered through 70-µm cell strainers. To determine thymocyte cellularity, Trypan Blue (Sigma-Aldrich, St. Louis, MO)-negative viable cells were counted on KOVA microscope slides (Hycor Biomedical, Garden Grove, CA). For in vitro stimulation, cells were cultured at 37°C at 5×10^6^ cells/ml in RPMI 1640 (Invitrogen, Carlsbad, CA), 5% heat-inactivated FBS (Invitrogen), 50 µM β-mercaptoethanol (Sigma-Aldrich) and stimulated with PMA and ionomycin (both from Sigma-Aldrich) at the indicated concentrations for the indicated period of time.

### Nuclear extract preparation and electrophoretic mobility shift assays (EMSA)

Thymocyte and leukemic cell nuclear extracts were prepared as described by Olnes and Kurl [56]. Briefly, 5×10^7^ cells were resuspended in 500 µl lysis buffer (10 mM Hepes [pH 7.9], 10 mM KCl, 0.1 mM EDTA, 0.1 mM EGTA, and 1 mM DTT) with protease and phosphatase inhibitors (1 mM phenylmethylsulphonylfluoride, 15 µg/ml aprotinin, 10 µg/ml leupeptin, 1 mM Na_3_VO_4_, 15 mM β-glycerolphosphate, and 10 mM *para*-nitrophenylphosphate; all from Sigma-Aldrich), rested for 10 min on ice, and lyzed using a Dounce homogenizer (25 strokes with a tight pestle) (Wheaton, Millville, NJ). The cellular lysate was pelleted at 110*g*, the supernatant removed, and nuclei were lyzed in 100 µl of extraction buffer (20 mM Hepes [pH 7.9], 400 mM NaCl, 1 mM EDTA, 1 mM EGTA, 10% glycerol, and 1 mM DTT) and protease and phosphatase inhibitors. The nuclear extracts were collected after centrifugation at 20,000*g*. EMSA were performed essentially as described [57], using Klenow polymerase (New England Biolabs, Ipswich, MA) end-labeled double-stranded oligonucleotide probes containing the Ig-κB NF-κB-binding site (plus strand: 5′-CAGAGGGGACTTTCCGAGAGG-3′) [58], the IL2Rα promoter palindromic NF-κB site (plus strand: 5′-TTGGCAACGGCAGGGGAATTCCCCTCTCCTTA-3′) [59], and Sp1-binding site (plus strand: 5′-ATTCGATCGGGGCGGGGCGAGC-3′). DNA binding reactions were carried out for 10 min at 0°C in a final volume of 16 µl containing 1 ng (∼125 fmol) of oligonucleotide probe, 10 mM HEPES [pH 7.4], 25 mM KCl, 1.25 mM Na phosphate, 175 µM EDTA, 75 µM EGTA, 1 mM DTT, 5 mM MgCl_2_, 1.5 µg poly[d(I-C)], 0.4 µg salmon sperm DNA and 1–5 µg nuclear extracts. For supershift analyses, polyclonal antibodies against p50 (n°71 and n°1263; kindly provided by John Hiscott and Nancy Rice, respectively), p52 (n°1495; kindly provided by Nancy Rice), and RelA (sc-109), RelB (sc-226), and c-Rel (sc-71) from Santa Cruz Biotechnology (Santa Cruz, CA) were incubated with binding reactions for 10 min on ice before electrophoresis.

### Flow cytometry

Single cell suspensions from lymphoid organs were stained with fluorochrome-labeled antibodies and detected by a FACSCalibur cytometer (BD Biosciences, San Jose, CA) as previously described [21]. Fluorescein isothiocyanate (FITC)-, R-phycoerythrin (PE)-, PE-cyanine 5 (PE-Cy5)- or allophycocyanin (APC)-conjugated antibodies specific for CD90.2/Thy1.2 (53-2.1) CD4 (H129.19), CD8α (53-6.7), CD3ε (145-2C11), TCRβ (H57-597), TCRγδ (GL3), CD5 (53-7.3), CD24/HSA (M1/69), and CD25/IL-2Rα (7D4) (BD Biosciences) were used. The data were analyzed using CellQuest (BD Biosciences) and FlowJo (Tree Star, Ashland, OR).

### Histopathological and immunohistochemical analyses

Histopathological analyses were performed essentially as previously described [21]. For immunohistochemistry, 6-µm formalin-fixed, paraffin-embedded, thymus sections were deparaffinized and hydrated. After each of the following incubations the slides were rinsed with several changes of PBS. Sections were blocked with 0.3% H_2_O_2_ in PBS for 15 min, followed by 0.5% casein in PBS for 15 min and incubated with 1∶50 biotinylated UEA-1 lectin for 2 h at room temperature followed by ABC peroxidase for 30 min. Sections were visualized by DAB staining, counterstained with Meyers hematoxylin, dehydrated and mounted in non-aqueous mounting media. All staining reagents were obtained from Vector Labs (Burlingame, CA).

## Supporting Information

Figure S1 TEL-JAK2 leukemic cells do not show c-Rel DNA-binding activity. (A) NF-κB activity (bands I and II, indicated by arrowheads) in nuclear extracts obtained from leukemic cells from a representative TEL-JAK2 tumor (n°20) does not include c-Rel, as shown by supershift analysis using the indicated antibodies. (B) The same c-Rel antibody supershifted an NF-κB complex (open arrowheads) in thymocytes stimulated for 24 h by 5 ng/ml PMA plus 250 ng/ml ionomycin.
(2.30 MB TIF)

Figure S2 TEL-JAK2;*Nfkb1* ^−/−^ leukemic cells activate RelA when stimulated with PMA plus ionomycin. (A) TEL-JAK2;*Nfkb1* ^−/−^ leukemic cells were stimulated for the indicated period of time with 50 ng/ml PMA plus 500 ng/ml ionomycin and nuclear extracts were analyzed by EMSA using the Ig-κB and Sp1 probes. (B) PMA plus ionomycin stimulation for 30 min induced RelA and p52. RelA-containing complexes are indicated by arrows. The p52:RelB complexes (band *II*) are shown by arrowheads.
(2.26 MB TIF)

Figure S3 Histological analysis of *Tcra* ^−/−^;*Relb* ^−/−^ and control mice. (A) H&E staining of liver (a–c) and lung (d–f) from mice of the indicated genotypes reveals an absence of inflammatory infiltrates in *Tcra* ^−/−^;*Relb* ^−/−^ mice. (B) H&E staining of mesenteric lymph nodes (a–c) from mice of the indicated genotypes shows rudimentary lymph nodes in *Relb*-deficient mice, as compared to *Relb*-proficient mice. Also note the presence of B-cell follicles in RelB-proficient lymph nodes (arrowheads).
(9.62 MB TIF)

Figure S4 TCRγδ T cells develop normally in *Tcra*;*Relb* double deficient thymi. TCRγδ and CD5 cell surface immunostaining of gated Thy1.2+, CD4/CD8 double negative cells of representative mice of the indicated genotypes.
(2.52 MB TIF)

Figure S5 NF-κB activity in TEL-JAK2 leukemic cells depends on αβTCR expression. (A) Leukemic cell nuclear extracts from representative TEL-JAK2;*Tcra* ^+/−^ and TEL-JAK2;*Tcra* ^−/−^ mice were analyzed by EMSA using the Ig-κB and Sp1 probes. (B) Antibody supershift analysis of Ig-κB-bound complexes using the indicated antibodies was performed on nuclear extracts from representative TEL-JAK2;*Tcra* ^−/−^ leukemic cells. *I* and *II* indicate migrating DNA-bound NF-κB complexes.
(1.95 MB TIF)

Figure S6 Diseased *Relb*-deficient and *Relb*-proficient TEL-JAK2 mice show similar macroscopic appearance. The thymus (a,b), spleen (c,d), and lung (e,f) of a representative mouse of each indicated genotype is shown. Note the massive invasion of the organs by leukemic cells. Arrowheads indicate areas of leukemic cell infiltration in the lungs.
(6.72 MB TIF)

Figure S7 *Relb*-deficient and *Relb*-proficient TEL-JAK2 leukemic cells present a similar cell surface marker phenotype. (a–f) Cell surface staining with CD4 and CD8 antibodies of wild-type thymocytes (a), two representative TEL-JAK2;*Tcra* ^−/−^;*Relb* ^+/−^ mice (b,c), and three representative TEL-JAK2;*Tcra* ^−/−^;*Relb* ^−/−^ mice (d–f). (g–j) Cell surface staining with CD24 or CD25 antibodies of two representative TEL-JAK2;*Tcra* ^−/−^;*Relb* ^+/−^ mice (top panels), and three representative TEL-JAK2;*Tcra* ^−/−^;*Relb* ^−/−^ mice (bottom panels).
(5.22 MB TIF)

Figure S8 *Relb* deficiency in the hematopoietic compartment does not affect leukemic cell accumulation in lymphoid organs. Diseased mice adoptively transferred with *Relb*-deficient TEL-JAK2;*Tcra* ^−/−^ bone marrow cells (TEL-JAK2;*Tcra* ^−/−^;*Relb* ^−/−^→WT) presented lymphoid organ tumors of similar weight as mice transferred with TEL-JAK2;*Tcra* ^−/−^;*Relb* ^+/−^ bone marrow (TEL-JAK2;*Tcra* ^−/−^;*Relb* ^+/−^→WT). Thymus and lymph node weights were plotted for each group mice. The number of analyzed mice is given between parentheses.→
(5.98 MB TIF)

Figure S9 Efficient thymocyte development in wild-type mice adoptively transferred with either *Tcra* ^−/−^;*Relb* ^+/−^ or *Tcra* ^−/−^;*Relb* ^−/−^ bone marrow cells. Top panels: CD4 and CD8 immunostaining shows a very low proportion of SP (host) thymocytes in representative pairs of recipient wild-type (WT) mice, indicating that the majority of thymocytes originated from donor hematopoietic cells given that TCRα deficiency blocks DP to SP transition. Bottom panels: CD25 and CD44 staining of gated CD4/CD8 DN cells of representative WT mice reconstituted with either *Tcra* ^−/−^;*Relb* ^+/−^ or *Tcra* ^−/−^;*Relb* ^−/−^ bone marrow. Note that the RelB mutation does not significantly affect early thymocyte development.
(6.52 MB TIF)

Figure S10 Normal double negative and double positive thymocyte development in bone marrow-reconstituted *Tcra* ^−/−^;*Relb* ^+/+^ and *Tcra* ^−/−^;*Relb* ^−/−^ mice. (A) CD4, CD8 cell surface immunostaining of total thymocytes (top panels) and CD25, CD44 staining of Thy1.2+, CD4/CD8 DN cells (bottom panels) of representative pairs of *Tcra* ^−/−^→*Tcra* ^−/−^;*Relb* ^−/−^ and *Tcra* ^−/−^→*Tcra* ^−/−^;*Relb* ^+/+^ chimeric mice. (B) Thymocytes from chimeric recipient male mice of the indicated genotypes that received bone marrow cells from female *Tcra* ^−/−^ donors were analyzed by PCR amplification of the *Jarid1c*/*Kdm5c* and *Jarid1d*/*Kdm5d* genes. The upper band derived from the *Jarid1c* gene, located on the X chromosome, while the lower band derived from the *Jarid1d* gene, located on the Y chromosome [55]. Note the full reconstitution of recipient male thymus with thymocytes of donor (female) origin.
(5.88 MB TIF)

Figure S11 TEL-JAK2-induced T-cell lymphoid tumors are smaller in *Tcra* ^−/−^;*Relb* ^−/−^ than *Tcra* ^−/−^;*Relb* ^+/−^ recipient mice. (A) Kaplan-Meier leukemia-free survival curves for *Tcra* ^−/−^;*Relb* ^−/−^ and *Tcra* ^−/−^;*Relb* ^+/−^ chimeric mice that received bone marrow from EμSRα-TEL-JAK2;*Tcra* ^−/−^ transgenic mice (median survival of 211 and 164 days, respectively; log-rank test, *P* value = 0.1016). The number of mice in each group is given between parentheses. (B) Thymus and lymph node weights were plotted for TEL-JAK2;*Tcra* ^−/−^→*Tcra* ^−/−^;*Relb* ^−/−^ and TEL-JAK2;*Tcra* ^−/−^→*Tcra* ^−/−^;*Relb* ^+/−^ chimeric mice that developed T-cell leukemia/lymphoma. **, *P* value<0.01; ***, *P* value<0.001 (unpaired *t*-test). The number of mice analyzed is given between parentheses.
(5.41 MB TIF)
