# Supplementary figures and images for: RelB-Dependent Stromal Cells Promote T-Cell Leukemogenesis
Source: PLoS One. 2008 Jul 2;3(7):e2555. doi: 10.1371/journal.pone.0002555 (PMC2440518; doi:10.1371/journal.pone.0002555)

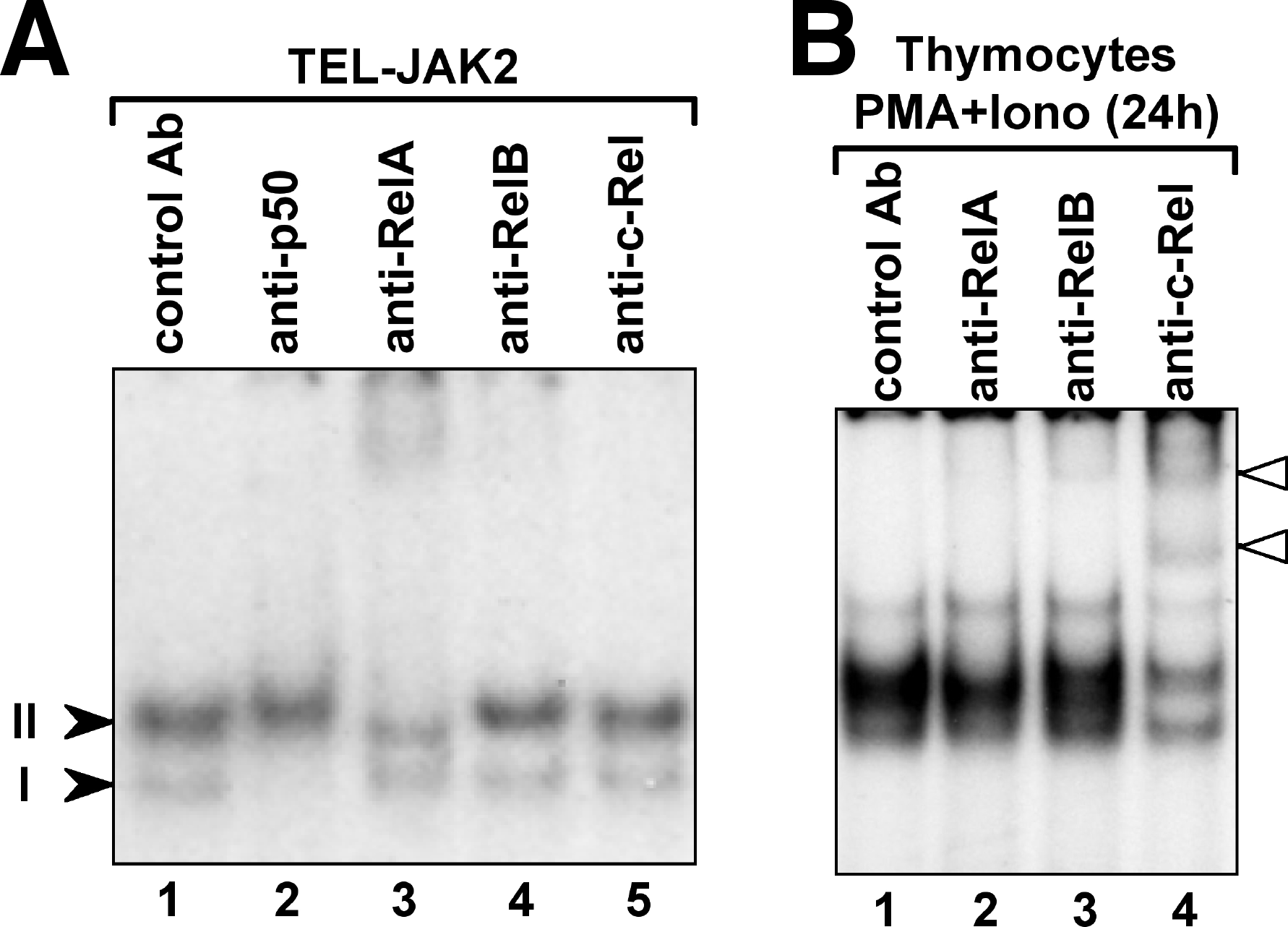

Supplement: Figure S1 — TEL-JAK2 leukemic cells do not show c-Rel DNA-binding activity. (A) NF-κB activity (bands I and II, indicated by arrowheads) in nuclear extracts obtained from leukemic cells from a representative TEL-JAK2 tumor (n°20) does not include c-Rel, as shown by supershift analysis using the indicated antibodies. (B) The same c-Rel antibody supershifted an NF-κB complex (open arrowheads) in thymocytes stimulated for 24 h by 5 ng/ml PMA plus 250 ng/ml ionomycin. (2.30 MB TIF) [file pone.0002555.s001.tif]

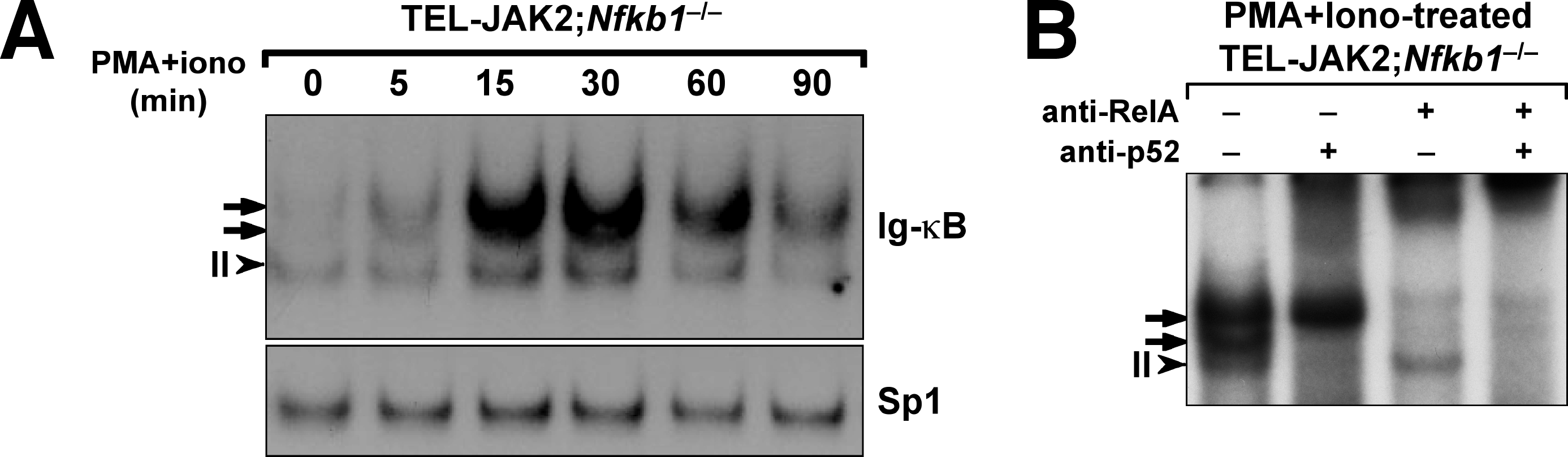

Supplement: Figure S2 — TEL-JAK2;Nfkb1 −/− leukemic cells activate RelA when stimulated with PMA plus ionomycin. (A) TEL-JAK2;Nfkb1 −/− leukemic cells were stimulated for the indicated period of time with 50 ng/ml PMA plus 500 ng/ml ionomycin and nuclear extracts were analyzed by EMSA using the Ig-κB and Sp1 probes. (B) PMA plus ionomycin stimulation for 30 min induced RelA and p52. RelA-containing complexes are indicated by arrows. The p52:RelB complexes (band II) are shown by arrowheads. (2.26 MB TIF) [file pone.0002555.s002.tif]

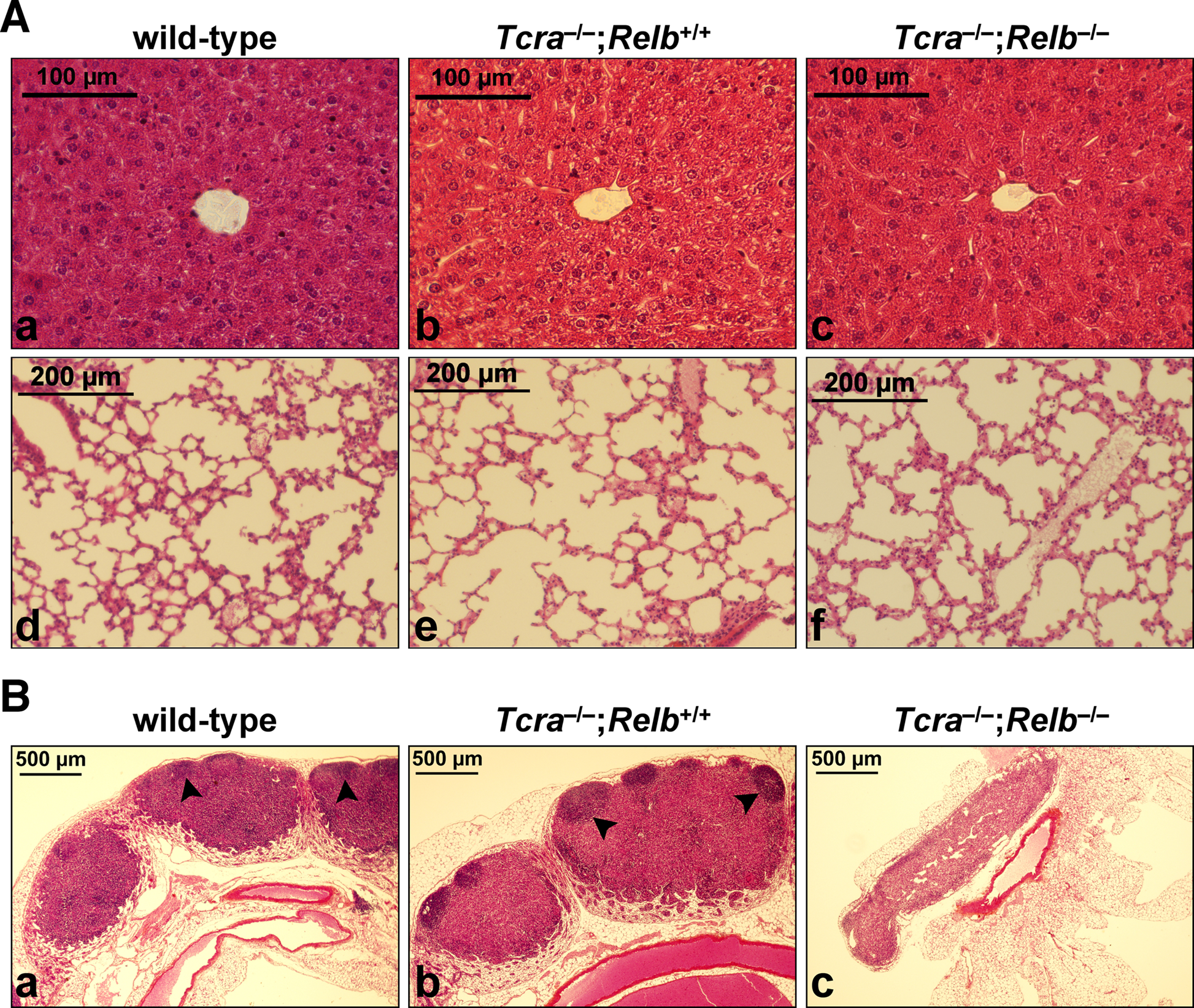

Supplement: Figure S3 — Histological analysis of Tcra −/−;Relb −/− and control mice. (A) H&E staining of liver (a–c) and lung (d–f) from mice of the indicated genotypes reveals an absence of inflammatory infiltrates in Tcra −/−;Relb −/− mice. (B) H&E staining of mesenteric lymph nodes (a–c) from mice of the indicated genotypes shows rudimentary lymph nodes in Relb-deficient mice, as compared to Relb-proficient mice. Also note the presence of B-cell follicles in RelB-proficient lymph nodes (arrowheads). (9.62 MB TIF) [file pone.0002555.s003.tif]

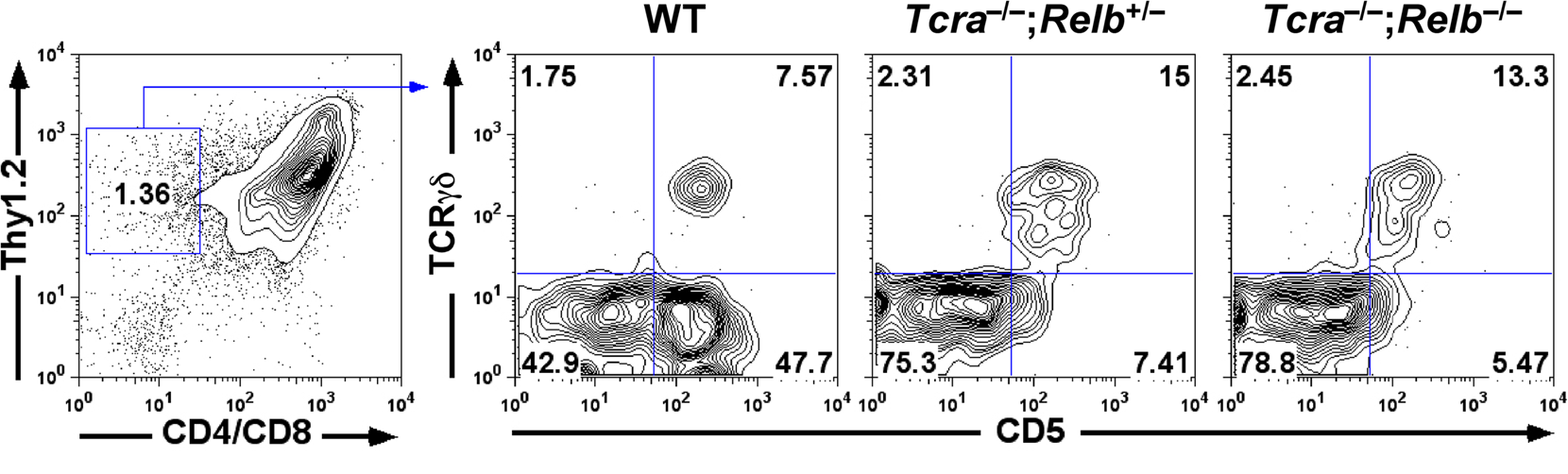

Supplement: Figure S4 — TCRγδ T cells develop normally in Tcra;Relb double deficient thymi. TCRγδ and CD5 cell surface immunostaining of gated Thy1.2+, CD4/CD8 double negative cells of representative mice of the indicated genotypes. (2.52 MB TIF) [file pone.0002555.s004.tif]

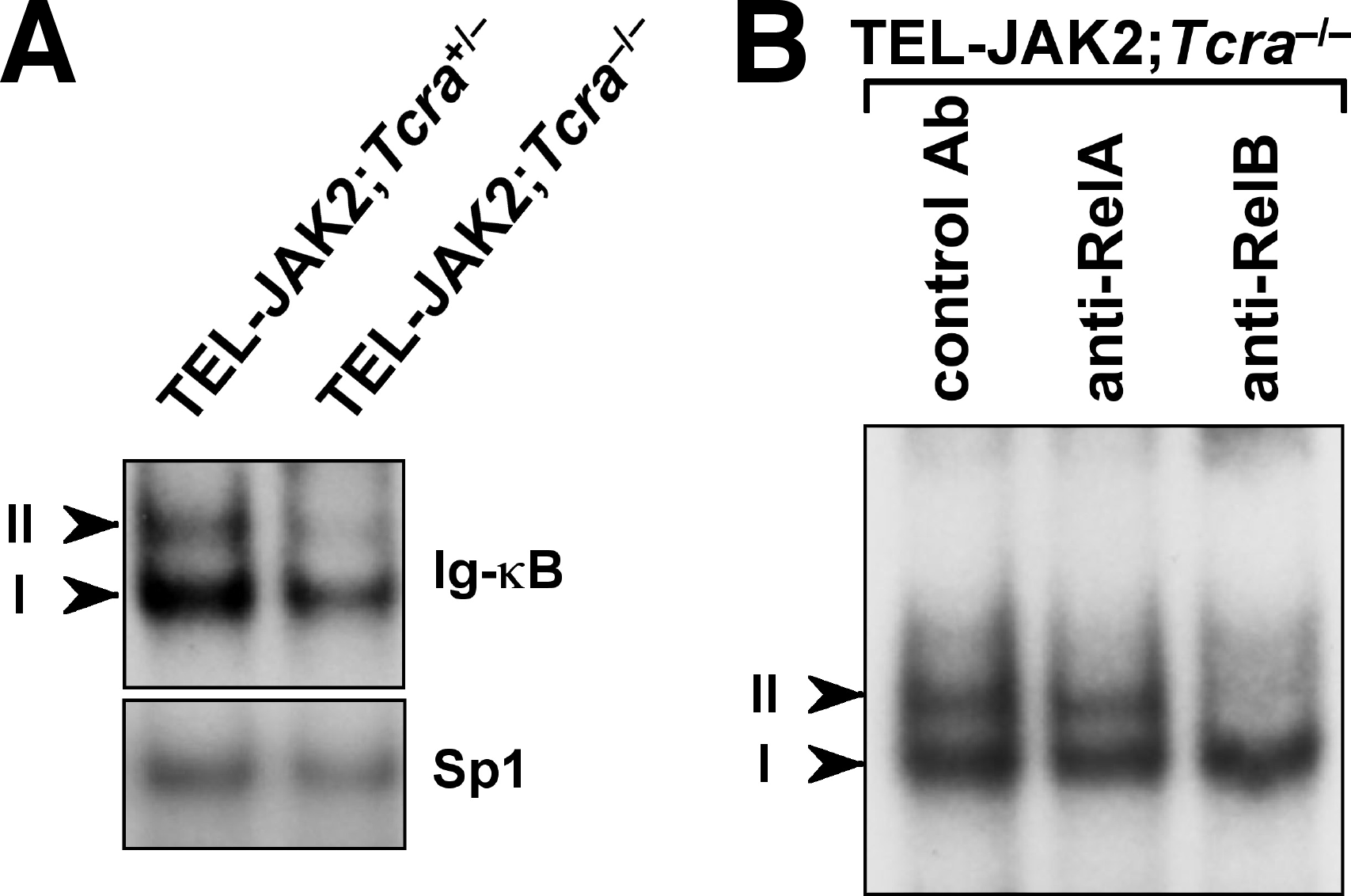

Supplement: Figure S5 — NF-κB activity in TEL-JAK2 leukemic cells depends on αβTCR expression. (A) Leukemic cell nuclear extracts from representative TEL-JAK2;Tcra +/− and TEL-JAK2;Tcra −/− mice were analyzed by EMSA using the Ig-κB and Sp1 probes. (B) Antibody supershift analysis of Ig-κB-bound complexes using the indicated antibodies was performed on nuclear extracts from representative TEL-JAK2;Tcra −/− leukemic cells. I and II indicate migrating DNA-bound NF-κB complexes. (1.95 MB TIF) [file pone.0002555.s005.tif]

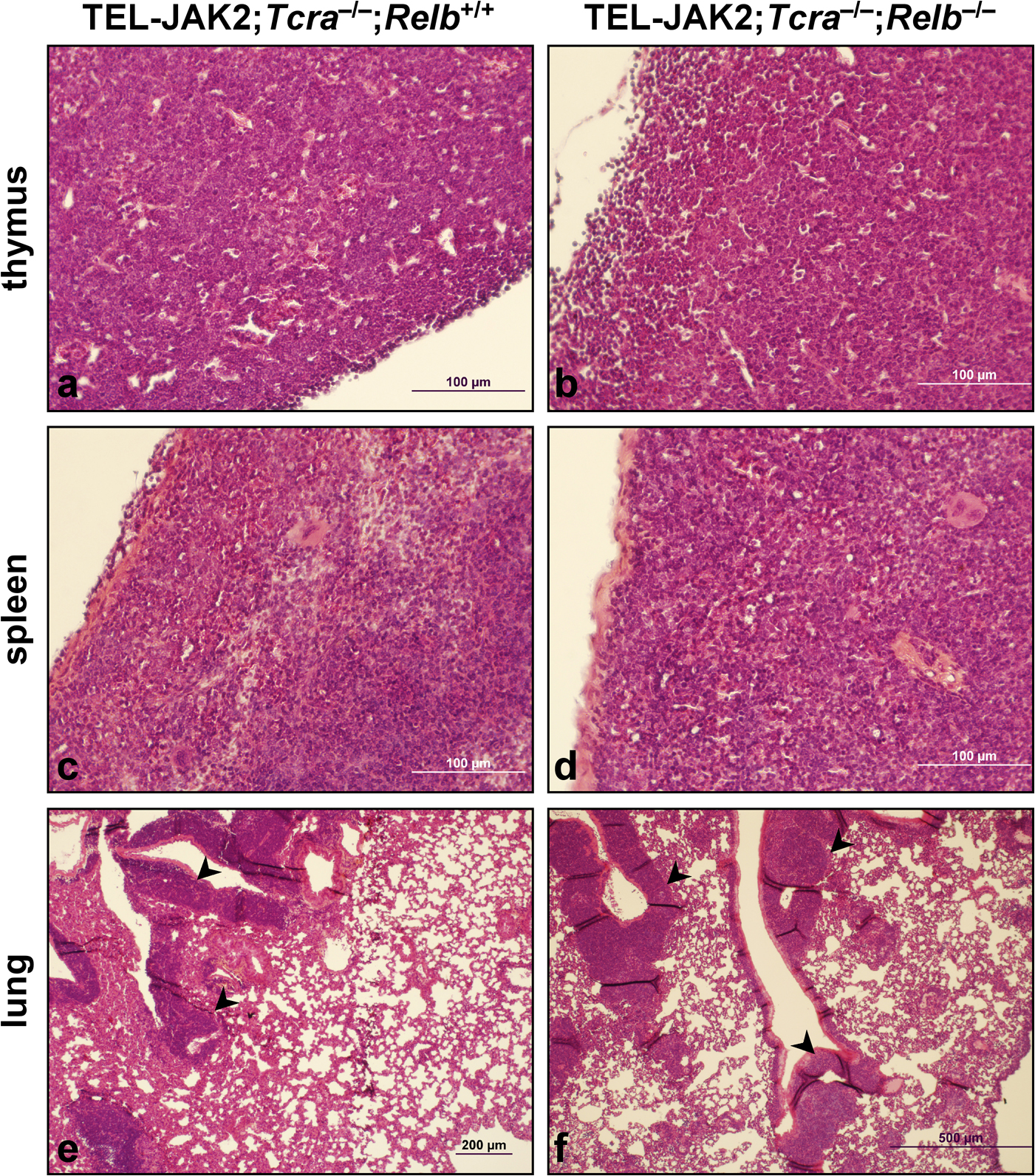

Supplement: Figure S6 — Diseased Relb-deficient and Relb-proficient TEL-JAK2 mice show similar macroscopic appearance. The thymus (a,b), spleen (c,d), and lung (e,f) of a representative mouse of each indicated genotype is shown. Note the massive invasion of the organs by leukemic cells. Arrowheads indicate areas of leukemic cell infiltration in the lungs. (6.72 MB TIF) [file pone.0002555.s006.tif]

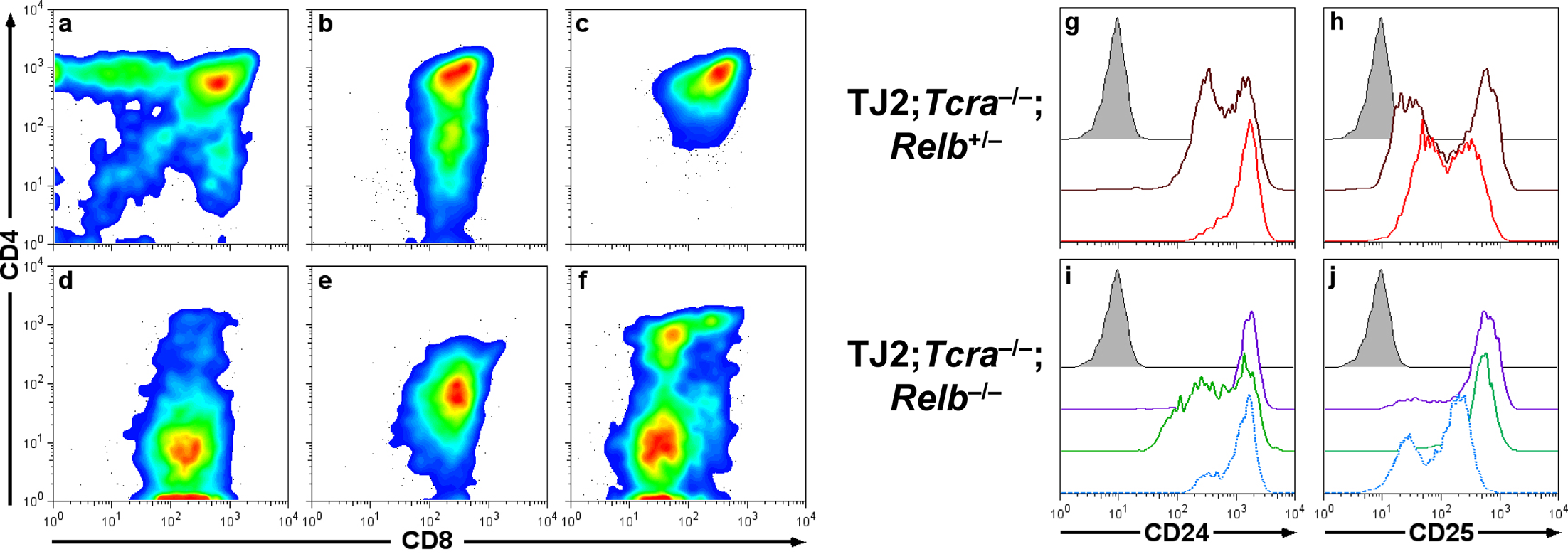

Supplement: Figure S7 — Relb-deficient and Relb-proficient TEL-JAK2 leukemic cells present a similar cell surface marker phenotype. (a–f) Cell surface staining with CD4 and CD8 antibodies of wild-type thymocytes (a), two representative TEL-JAK2;Tcra −/−;Relb +/− mice (b,c), and three representative TEL-JAK2;Tcra −/−;Relb −/− mice (d–f). (g–j) Cell surface staining with CD24 or CD25 antibodies of two representative TEL-JAK2;Tcra −/−;Relb +/− mice (top panels), and three representative TEL-JAK2;Tcra −/−;Relb −/− mice (bottom panels). (5.22 MB TIF) [file pone.0002555.s007.tif]

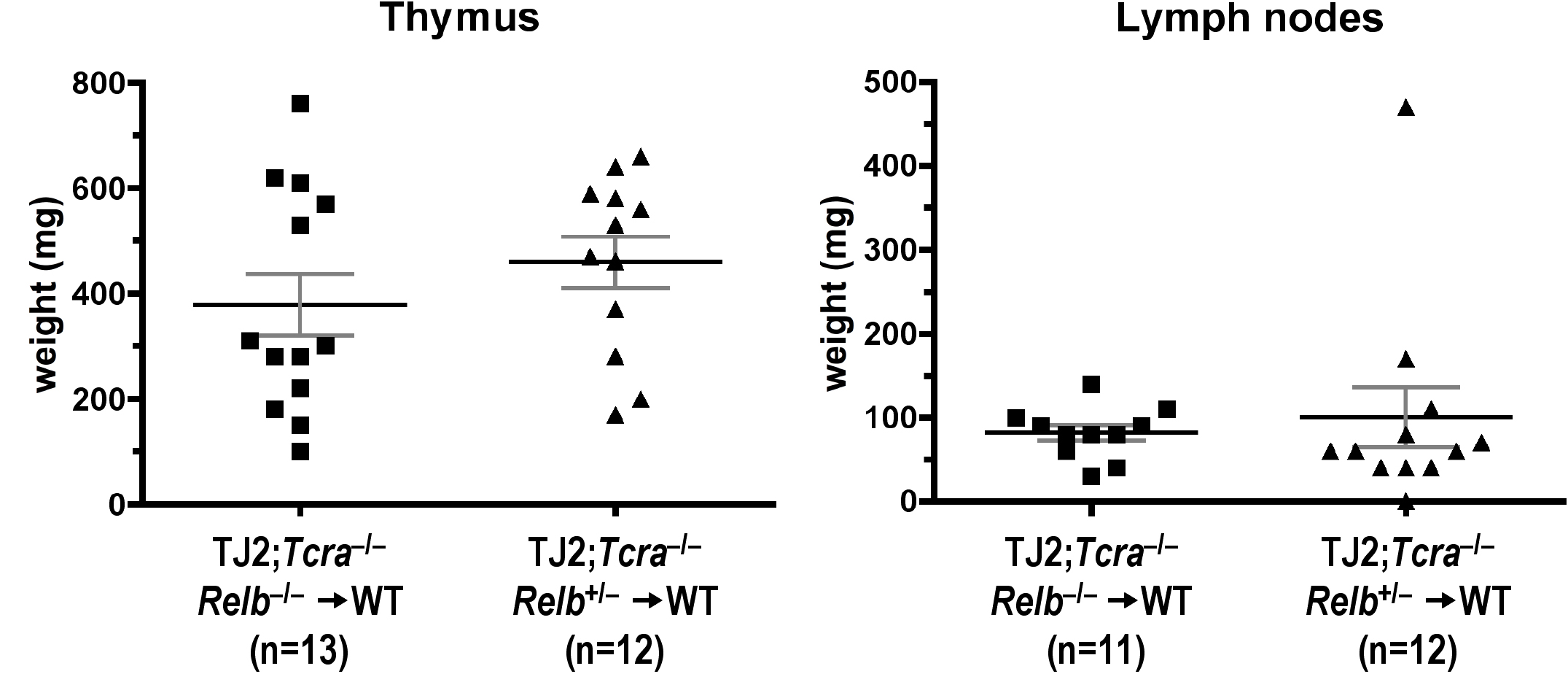

Supplement: Figure S8 — Relb deficiency in the hematopoietic compartment does not affect leukemic cell accumulation in lymphoid organs. Diseased mice adoptively transferred with Relb-deficient TEL-JAK2;Tcra −/− bone marrow cells (TEL-JAK2;Tcra −/−;Relb −/−→WT) presented lymphoid organ tumors of similar weight as mice transferred with TEL-JAK2;Tcra −/−;Relb +/− bone marrow (TEL-JAK2;Tcra −/−;Relb +/−→WT). Thymus and lymph node weights were plotted for each group mice. The number of analyzed mice is given between parentheses.→ (5.98 MB TIF) [file pone.0002555.s008.tif]

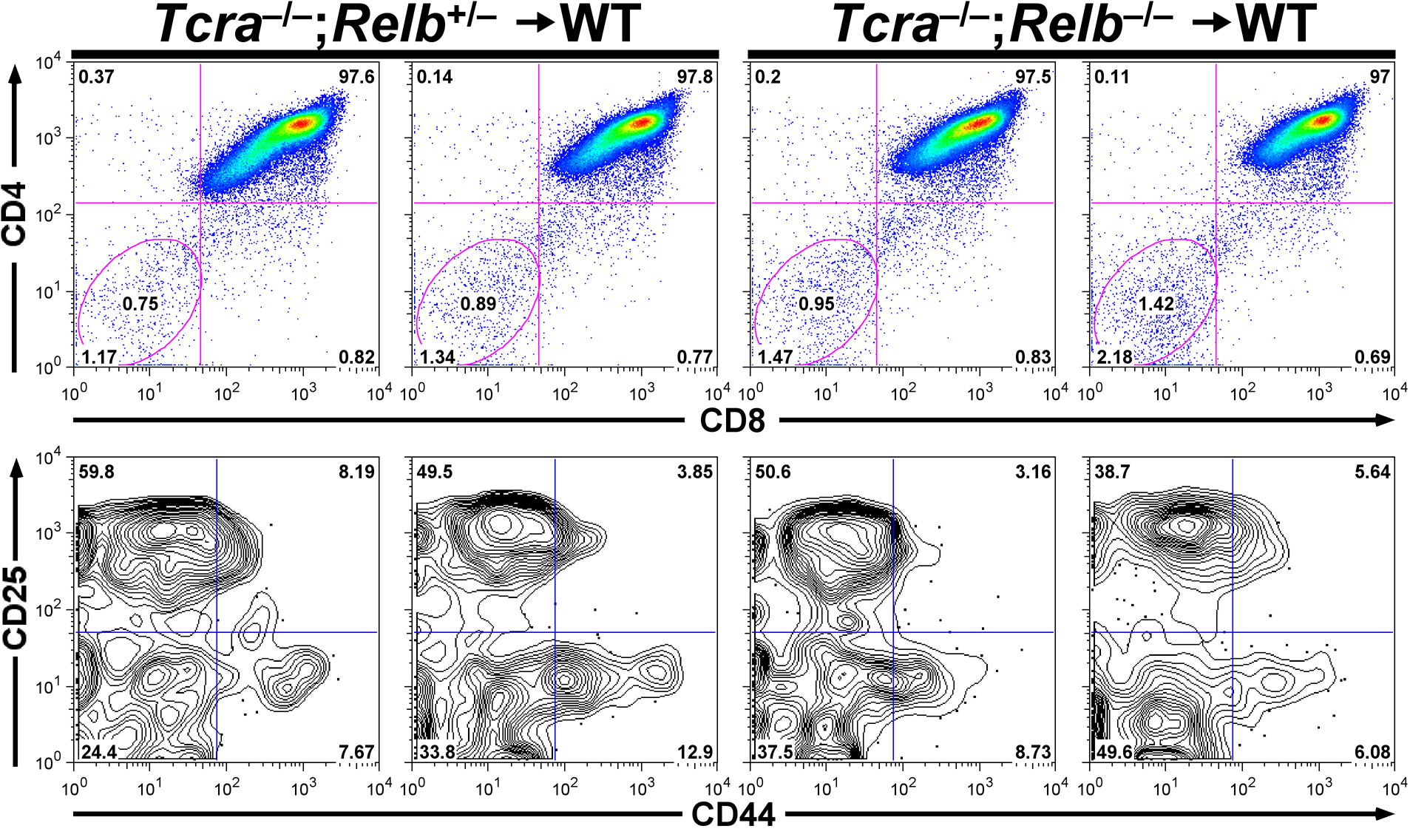

Supplement: Figure S9 — Efficient thymocyte development in wild-type mice adoptively transferred with either Tcra −/−;Relb +/− or Tcra −/−;Relb −/− bone marrow cells. Top panels: CD4 and CD8 immunostaining shows a very low proportion of SP (host) thymocytes in representative pairs of recipient wild-type (WT) mice, indicating that the majority of thymocytes originated from donor hematopoietic cells given that TCRα deficiency blocks DP to SP transition. Bottom panels: CD25 and CD44 staining of gated CD4/CD8 DN cells of representative WT mice reconstituted with either Tcra −/−;Relb +/− or Tcra −/−;Relb −/− bone marrow. Note that the RelB mutation does not significantly affect early thymocyte development. (6.52 MB TIF) [file pone.0002555.s009.tif]

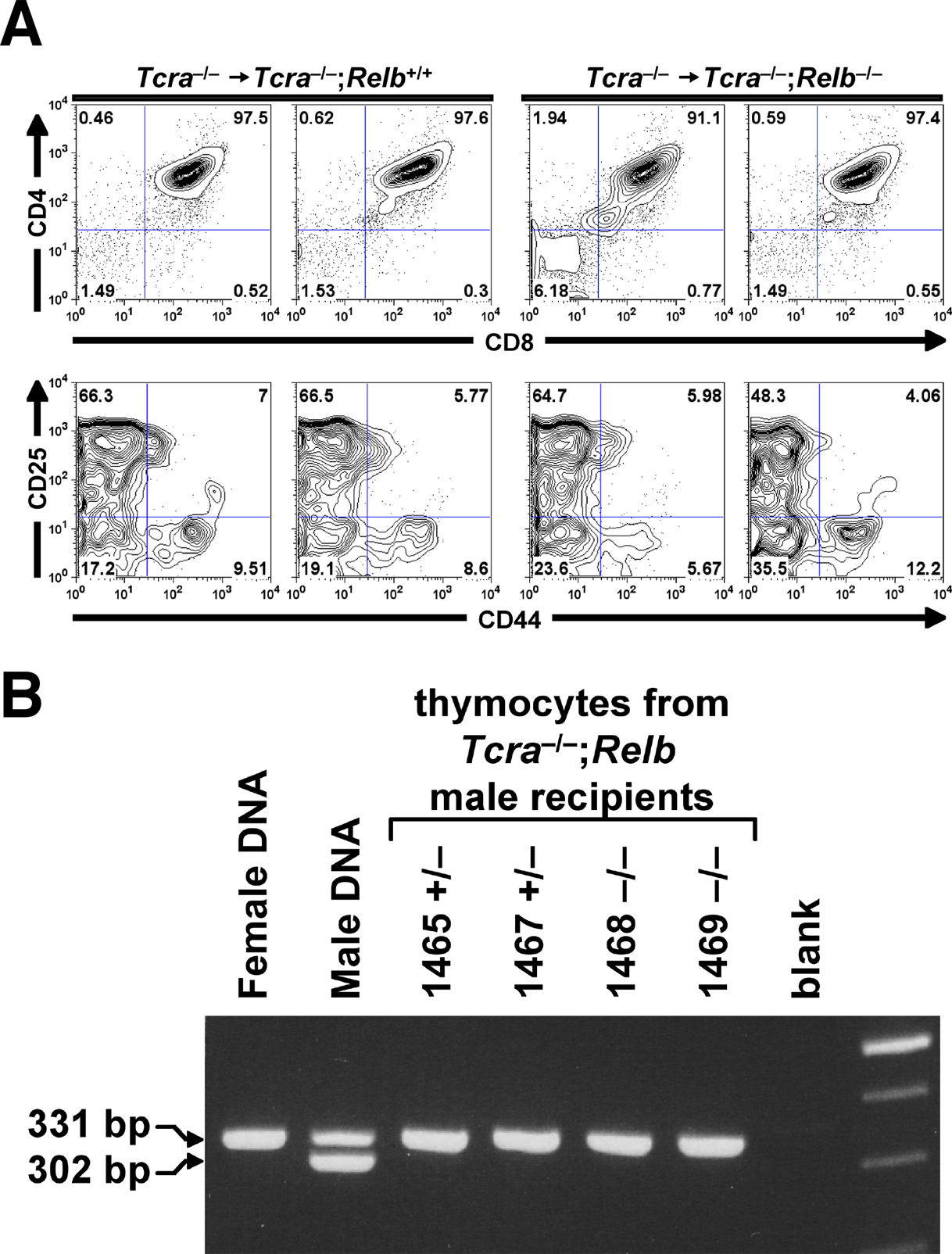

Supplement: Figure S10 — Normal double negative and double positive thymocyte development in bone marrow-reconstituted Tcra −/−;Relb +/+ and Tcra −/−;Relb −/− mice. (A) CD4, CD8 cell surface immunostaining of total thymocytes (top panels) and CD25, CD44 staining of Thy1.2+, CD4/CD8 DN cells (bottom panels) of representative pairs of Tcra −/−→Tcra −/−;Relb −/− and Tcra −/−→Tcra −/−;Relb +/+ chimeric mice. (B) Thymocytes from chimeric recipient male mice of the indicated genotypes that received bone marrow cells from female Tcra −/− donors were analyzed by PCR amplification of the Jarid1c/Kdm5c and Jarid1d/Kdm5d genes. The upper band derived from the Jarid1c gene, located on the X chromosome, while the lower band derived from the Jarid1d gene, located on the Y chromosome [55]. Note the full reconstitution of recipient male thymus with thymocytes of donor (female) origin. (5.88 MB TIF) [file pone.0002555.s010.tif]

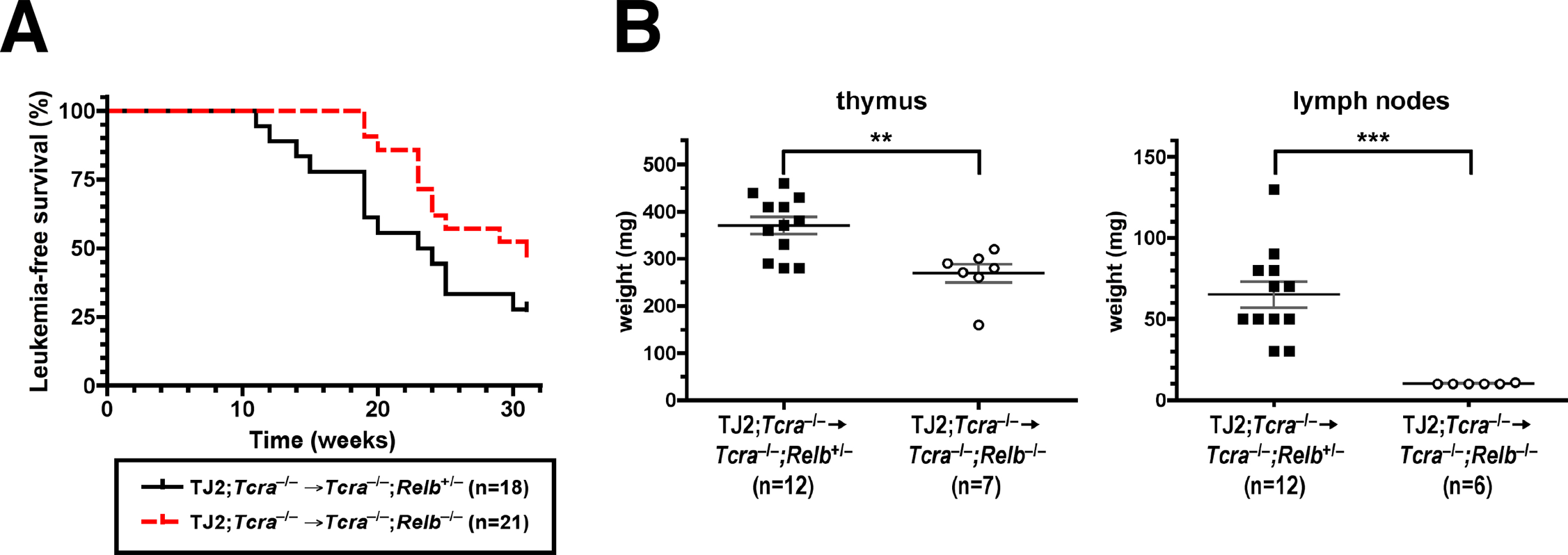

Supplement: Figure S11 — TEL-JAK2-induced T-cell lymphoid tumors are smaller in Tcra −/−;Relb −/− than Tcra −/−;Relb +/− recipient mice. (A) Kaplan-Meier leukemia-free survival curves for Tcra −/−;Relb −/− and Tcra −/−;Relb +/− chimeric mice that received bone marrow from EμSRα-TEL-JAK2;Tcra −/− transgenic mice (median survival of 211 and 164 days, respectively; log-rank test, P value = 0.1016). The number of mice in each group is given between parentheses. (B) Thymus and lymph node weights were plotted for TEL-JAK2;Tcra −/−→Tcra −/−;Relb −/− and TEL-JAK2;Tcra −/−→Tcra −/−;Relb +/− chimeric mice that developed T-cell leukemia/lymphoma. **, P value<0.01; ***, P value<0.001 (unpaired t-test). The number of mice analyzed is given between parentheses. (5.41 MB TIF) [file pone.0002555.s011.tif]
